# Feedback based on simple strategies facilitates strategy execution and selection in foraging

**DOI:** 10.3758/s13423-025-02733-2

**Published:** 2025-08-04

**Authors:** Hsuan-Yu Lin, Bettina von Helversen

**Affiliations:** Department of Psychology, Faculty of Human and Health Sciences, University of Bremen, Hochschulring 18, 28359 Bremen, Germany

**Keywords:** Patch-leaving, Foraging, Decision-making, Computational modeling

## Abstract

Previous research has shown that human participants performed suboptimally in patch-leaving behavior during foraging tasks. This suboptimal performance stemmed from two primary sources: Participants often adopted a strategy unsuited to the environment and failed to apply it optimally. The current study investigates whether providing feedback on participants’ patch-leaving behavior can improve their performance by facilitating either a switch to a more effective strategy or an enhanced application of their existing strategy. All participants completed a patch-leaving task across three sessions: pre-feedback, feedback, and post-feedback. Their patch-leaving strategies in each session were identified through computational modeling. During the feedback session, participants received feedback based on either the fixed-time (FT) or giving-up-time (GUT) strategy. Most participants employed the GUT strategy in the pre-feedback session and showed improved performance in the post-feedback session. In the FT feedback condition, many participants switched to using the FT strategy in the post-feedback session. Participants who switched improved in performance, whereas those who continued using the GUT strategy did not. In contrast, in the GUT feedback condition, most participants continued using the GUT strategy but benefited from feedback due to a more precise execution of the GUT strategy in the post-feedback session. These results suggest that participants can adapt to a better-suited strategy or improve their application of a suboptimal strategy with appropriate feedback.

To evaluate if a coin is biased, one must consider the number of tosses before drawing conclusions. With each toss, information about the coin’s fairness increases, but not at a consistent rate. The initial tosses provide significant insights, while subsequent ones yield less information. Thus, after multiple tosses, when the gained information is minimal, it is more appropriate to make a decision rather than continue tossing. Different theories, whether through Bayesian or frequentist statistics, also influence how the stopping criterion is determined. What would happen if a Bayesian statistician were supervised by a frequentist? Would the Bayesian switch to use frequentist statistics or ignore the supervision?

This concept mirrors patch-leaving behavior in foraging. A forager enters a food-concentrated patch and gradually depletes its resources, reducing foraging efficiency. The forager must determine the optimal time to leave the patch to maintain overall foraging efficiency. The Marginal Value Theorem (MVT; Charnov, 1976) is generally viewed as the optimal approach for patch-leaving tasks. It suggests that a forager should remain in the current patch until its instantaneous reward rate falls below the environment’s average. Once this condition is met, staying in the current patch becomes inefficient, and the forager should leave. Prior studies have shown that humans tend to follow the optimal patch-leaving policy qualitatively (Pacheco-Cobos et al., 2019; Wolfe, 2013; Constantino & Daw, 2015). For instance, participants stay longer in rich patches compared to poor ones (Lin & von Helversen, 2023). However, people often leave too early or too late, rarely performing on an optimal level (Zhang, Gong, Fougnie, & Wolfe, 2015, 2017). This sub-optimal behavior is not surprising, as performing optimally requires almost complete knowledge of the environment and the current patch (Green, 1980, 1984; McNair, 1982). Instead, participants usually employ simplified heuristics to approximate the optimal behavior (Bettinger & Grote, 2016; Pyke, 1978; van Alphen, Bernstein, & Driessen, 2003).

## Patch-leaving strategies

There are several common strategies people employ in foraging: giving-up time (GUT), fixed-number (FN), and fixed-time (FT). According to the GUT strategy (McNair, 1982), the forager remains in a patch until no reward is obtained for a certain duration, i.e., the giving-up time. While imperfect, the continuous duration of no reward correlates with the current reward rate, leading foragers to leave patches when the current reward rate falls below a threshold, resembling the MVT. Foragers using the FN strategy leave the current patch after acquiring a fixed number of rewards from the patch. According to the FT strategy (Krebs, 1973) the forager departs after spending a predetermined amount of time in the patch. Both FN and FT strategies approximate MVT by assuming that the current reward rate decreases as the forager acquires more reward or spends more time in the patch. Thus, foragers using FN or FT also leave when the current reward rate falls below a threshold, similar to the MVT.

There are two reasons why participants perform suboptimally. Firstly, participants may underperform due to the selection of suboptimal strategies. The efficacy of the GUT, FN, and FT strategies depends on the variance of patch quality in the environment (Lin & von Helversen, 2023). The GUT strategy can adjust leaving times based on patch quality. Due to the correlation between the current reward rate and the continuous time of getting no reward, the GUT strategy generally stays longer in a high-quality patch, aligning with the MVT. In contrast, FN and FT strategies are inflexible regarding patch quality, leaving after the same number of acquired rewards or at the same time, regardless of quality. Consequently, GUT typically outperforms FN and FT in volatile environments with significant patch quality variation (Hutchinson, Wilke, & Todd, 2008; Lin & von Helversen, 2023). However, the FN and FT strategies tend to outperform the GUT strategy when the environment is relatively stable. Under a stable environment, where all the patches have a similar quality, the optimal reward acquired or the optimal leaving time are similar across patches. Hence, the FN and FT strategy can perform close to optimal. Comparing between FN and FT, simulation shows that FN is more susceptible to variance in the environment compared to FT.^1^ In contrast, the GUT strategy only leaves once it has stayed for a certain time without getting a reward. It thus always wastes precious time without getting a reward, preventing it from performing as closely to the optimal policy as the FT or FN strategy.

Accordingly, participants may achieve better performance if they choose the GUT strategy when patch qualities are variable but select the FN or FT strategy when patch qualities are similar. However, previous research has shown that participants often prefer the GUT strategy over the other strategies, even in environments where the FN or FT strategy performs better. For instance, Hutchinson et al. (2008) found that participants’ leaving behavior was primarily correlated with the giving-up-time, despite there being no variance in patch qualities (i.e., the FN strategy was the optimal strategy). Similarly, Lin and von Helversen (2023) reported that most participants were best described by the GUT strategy, regardless of patch quality variation, with only a few following the FT strategy. Almost no participants used the FN strategy.

Secondly, participants may sometimes perform suboptimally when using the GUT strategy, as they are best described by the GUT strategy but do not achieve the optimal performance level of GUT. For example, in Lin and von Helversen (2023), participants who employed the GUT strategy performed below the optimal GUT level in environments with moderate patch variation. Similarly, Hutchinson et al. (2008), reported participants performing suboptimally under no or high variance in patch qualities. The results are, however, inconsistent as in the study by Lin and von Helversen (2023) participants’ performance was close to optimal performance when there was no variance in patch qualities, as well as in the condition with moderate variation environment in the study by Hutchinson et al. (2008).

The goal of this paper is to investigate how participants’ performance can be enhanced, focusing on their ability to select effective strategies and improve their execution through targeted feedback.

Why participants do not switch to the FT strategy even when it is the better strategy remains unclear. One possible explanation is that the GUT strategy is treated as a default strategy by the participants, as the GUT strategy can perform relatively well regardless of patch quality variance (Lin & von Helversen, 2023). Indeed, a simulation study found that the GUT strategy maintains stable performance over a wide range of patch quality variance. In contrast, the FT strategy performs better under low-variance environments but experiences a sharp decline in performance as variance increases. Thus, the GUT is a safe strategy if participants are unaware of the environment. This applies to most patch-leaving research, as usually participants have to learn about the environment through experience (Hutchinson et al., 2008; Wilke, Hutchinson, Todd, & Czienskowski, 2009; Lin & von Helversen, 2023). Thus, it is possible that participants used the GUT strategy as a default option because they did not acquire enough knowledge of the current environment. Additionally, most of the patch distributions in ecological environments contain high variation (Kretzschmar & Adler, 1993), which also reinforces the GUT strategy as a default strategy.

One method of acquiring knowledge about an environment is through experiencing it. However, previous research has not demonstrated that participants are more likely to choose the optimal strategy with increased experience (Lin & von Helversen, 2023). Participants consistently preferred the GUT strategy in both the first and second halves of the task, even when patch quality remained constant (Lin & von Helversen, 2023). However, other studies have shown that feedback can impact participants’ performance and strategy selection (Evans & Brown, 2017; Hassall & Krigolson, 2020; Newell & Rakow, 2007; Starns & Ratcliff, 2010). For example, in a spatial foraging task, Hassall and Krigolson (2020) asked participants to search for rewards by sampling various locations on a map. Each sample yielded some reward, with the magnitude depending on the underlying reward distribution, which consisted of either one peak spot or multiple peak spots. Hassall and Krigolson (2020) informed the participants about the upcoming reward distribution and found that their search strategy changed based on the provided information. Participants searched more frequently when there were multiple patches in the upcoming area compared to only a single patch. Studying speed–accuracy trade-offs Evans and Brown (2017) asked participants to perform a motion discrimination task in which they judged the direction of moving dots. Participants were instructed to perform as many correct responses as possible within the allotted time frame. To achieve optimal performance, participants had to balance response speed and accuracy. In each trial, participants received feedback regarding the correctness of their responses. In addition, after each block, some participants received performance feedback and some participants received performance feedback and cognitive feedback about how to adjust their response speed to reach the optimal performance. Evans and Brown (2017) found that all participants improved, even participants who received no performance or cognitive feedback. These were, however, outperformed by participants receiving performance feedback and those with both performance and cognitive feedback, who performed best.

In this study, we follow Evans and Brown (2017) approach to examine if providing cognitive feedback about leaving patches too early or late improves participants’ performance after each leaving decision. Additionally, we compare the effectiveness of feedback based on the GUT versus FT strategy. Initially, participants performed the patch-leaving task without feedback. Subsequently, one group received feedback based on the GUT strategy and another received feedback based on the FT strategy. Finally, a third session was conducted to assess whether the participants had learned from the feedback.

Feedback based on the FT strategy might prompt participants to switch to an FT strategy because they learn (1) when to leave the patch and (2) that adhering to the feedback leads to good performance. Consequently, we hypothesized that participants would be more likely to rely on the FT strategy after receiving FT feedback. However, feedback based on a strategy other than the one used by participants can also be confusing, especially if they fail to recognize the crucial factor that the strategy is based on, i.e., time in the patch for the FT strategy. As such, for some participants using the GUT strategy, FT feedback may be confusing and provide no benefits from the feedback.

If participants fail to switch strategies, it might be easier for those using the GUT strategy to improve performance through GUT feedback. This feedback may help them execute the GUT strategy more closely to its optimal threshold. Consequently, we hypothesized that participants who used the GUT strategy in the first phase and received GUT feedback in the second phase will also see improvements in their performance.

## Experiment

To investigate these questions, we conducted an experiment with an experimental foraging task. Since we focused on the strategy selection by the participants, ideally, we want to set up the environment that is neutral to most strategies. However, since previous studies showed that most of the participants would use the GUT strategy regardless of whether the GUT strategy is optimal, we were particularly interested in whether we could induce participants to use the FT strategy. Thus, in this experiment, we set up an environment that is slightly advantageous for the FT strategy to encourage the participant to adopt the FT strategy.^2^

**Fig. 1:**
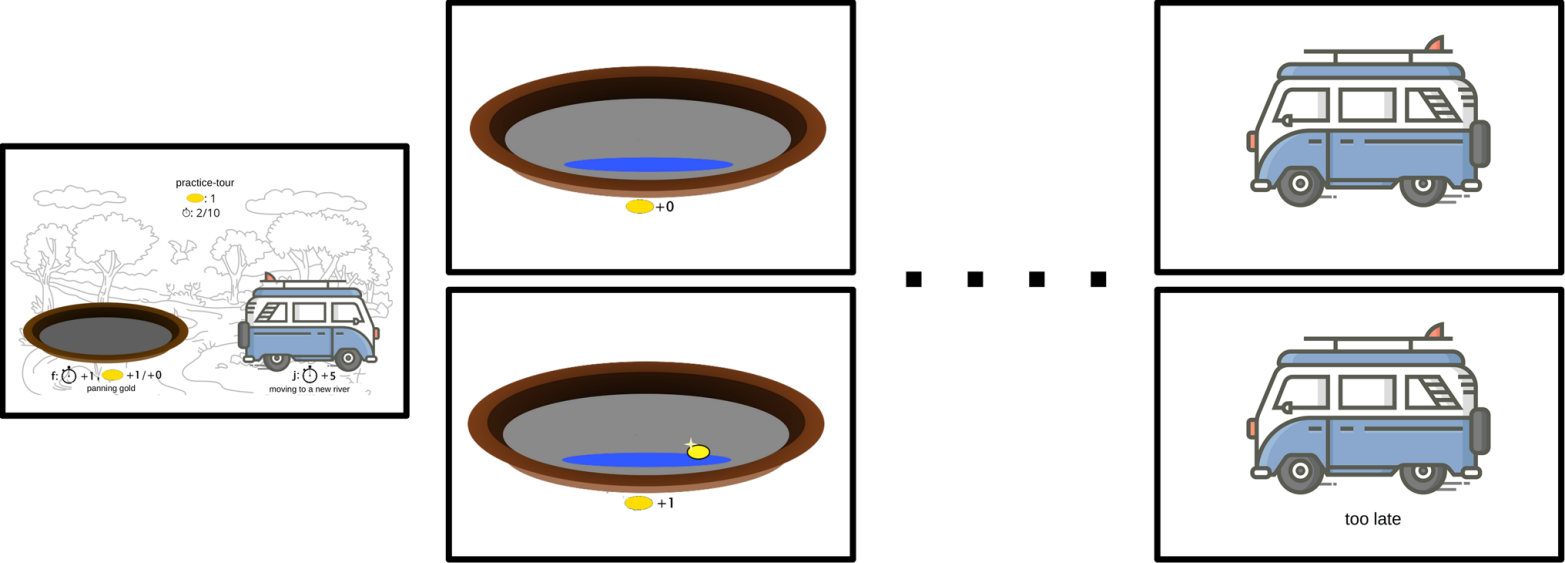
The procedure of each river. *Note.* The figure illustrates the experimental procedure. The leftmost screenshot shows the decision options presented to participants at a river where they could either pan gold or travel to a new river. On top of the screen, participants are informed about the accumulated gold at the current session and the time units they have spent. The bottom shows the two options for the participants, which they can select by pressing the respective key (f or j). If they decide to pan gold, participants are shown one of two animations, illustrated in the middle. The *middle top panel* shows the panning result "no gold", and the *middle bottom panel* the panning result "gold". If the participants decide to leave the current river, they are shown an animation of a moving car. If the current session consists of feedback, the feedback is displayed together with the animation (*bottom right*). Otherwise, there is no feedback displayed (*top right*). All the texts were originally in German and were translated into English in the figure

### Method

#### Participants

One hundred and fifteen students at the University of Bremen were recruited online, with a mean age of 25.27 years (range, 18–45) randomly assigned into one of the two feedback conditions. Participants were unaware of which condition they were assigned to. The reported gender distribution was 15 male, 98 female, and one diverse. One participant did not report their gender. Participants were awarded one course credit for participating in the experiment. Three participants were excluded from the analysis due to low performance (performance lower than 50% of the optimal performance) in pre-feedback session. An additional three participants were excluded from the analysis due to self-reported low involvement in the post-experiment questionnaire. All participants were given and agreed to a consent form informing them about experimental procedures, safety, and data security before starting the experiment. The experiment was conducted according to the APA ethical guidelines.

#### Material, procedure, and design

The experiment was conducted online and required an average of 40 min to complete. The experiment was programmed in JavaScript with the jsPsych 6.1 library (De Leeuw, 2015). The experiment was framed as a gold-panning tour, and participants were instructed to obtain as many gold nuggets as possible. The experiment was separated into three sessions, and each session consisted of 250 time units. In each session, participants started out at a river and could choose one of two actions by pressing either the f or j key on their keyboard: panning gold (f key) or moving to a new river (j key). Panning gold cost one time unit, and traveling to a new river cost five time units. If participants chose to pan gold, a short animation played to inform the participant about the outcome of the panning, either a gold nugget or nothing. If participants chose to move to a new river, a short moving animation played, indicating movement. Regardless of which actions participants chose to take, their remaining time units and the number of gold nuggets acquired were updated after the animation ended. Participants were then able to choose their subsequent action. This process was repeated until participants spent all the time units.

**Table 1:** Summary of the strategy’s behavior under optimal performance

Model	Optimal strategy parameter	Optimal performance	Average resident time		
			River quality		
			5	8	11
MVT	0.236 (reward rate)	58.96	4.99	13.67	19.73
GUT	4 (giving-up-time)	51.68	7.99	10.84	14.41
FT	12 (resident time)	54.02	12	12	12
FN	3 (number of rewards)	49.51	15.71	8.69	6.04

Strategy abbreviations: MVT, marginal value theorem; GUT, giving-up-time strategy; FT, fixed-time strategy; FN, fixed-number strategy. The ’optimal strategy parameter’ denotes the best-performing parameter given a certain strategy. For the MVT strategy, the parameter is the momentary reward rate; for the GUT strategy, the parameter is the continuous time of receiving no reward; for the FT strategy, the parameter is the resident time; for the FN strategy, the parameter is the number of rewards received within the patch. The optimal performance is the average number of rewards acquired for a session when performing optimally

The three sessions were *pre-feedback*, *feedback*, and *post-feedback* session, and the participants experienced the three sessions in consecutive order. The *pre-feedback* session was identical to the procedure described earlier. Before the *feedback* session, the participants were informed that an instructor would join the tour with them. After the participants decided to leave a river, the instructor informed the participants regarding their timing in leaving the river. The instructor would provide either *too late*, *too early*, or *right on time* feedback depending on the participants’ leave time and the leave time when executing a strategy optimally. The strategy used by the instructor was either the GUT strategy or the FT strategy, and the strategy was manipulated between participants. The participants were not informed about the strategy used by the instructor during the experiment. After the *feedback* session, the participants were informed that the instructor was sick and could not join the next session, and the *post-feedback* session proceeded identical to the *pre-feedback* session (Fig. 1).

Before the experimental sessions, participants performed a practice session. The practice session followed the same procedures as the experimental sessions, except that it consisted of only 20 time units and no feedback was provided. After the practice session, participants were asked to answer two quiz questions regarding the time spent traveling to a new river and their goal in the experiment. If participants failed to answer the questions correctly, they were asked to re-read the instructions and redo the practice session. After the experimental sessions, participants were asked to fill in a post-experiment questionnaire regarding their involvement in the experiment. Participants were asked to complete the questionnaire honestly and were informed that their answers would not affect their eligibility for course credits. Participants who reported low involvement still received course credit but were removed from the analysis.^3^

The probability of finding a gold nugget at any time unit was related to the number of gold nuggets remaining at the current river, and the probability is $N_{remain} \times 0.05$. Participants were informed about the relationship between the probability of finding a nugget and the number of nuggets remaining in the patch. The rivers contained either 5, 8, or 11 gold nuggets initially across all the sessions. The three different river qualities were encountered with equal probability, and the experimental program would attempt to balance the presence of all three river qualities. There was no indication regarding the current river quality other than experiencing the rate of finding gold nuggets, and the participants were only informed that the quality of rivers could vary. Under the distribution of the river qualities, the FT strategy outperforms the GUT strategy by 4.5%. Table 1 summarizes the performance of the strategies when they are executed optimally, i.e., the decision parameter of the strategy (i.e., momentarily reward rate for MVT or the number of time units without a reward for the GUT strategy etc.) is selected to maximize its performance in the task.

### Result

Because the *feedback* session differed procedurally from the *pre-feedback* and *post-feedback* sessions, and the mere presence of feedback might influence participants’ behavior during this session, we excluded the data from the *feedback* session in our analysis. However, we included participants’ behavior during the *feedback* session in Table 2 for completeness. We defined performance as the total amount of gold acquired within a session, excluding the last river if the session ended without leaving it.^4^ We analyzed the remaining data using the BayesFactor package (Morey & Rouder, 2015) in R (R Core Team, 2024). First, we conducted a Bayesian analysis of variance with factors sessions (within participants) and feedback type (between participants), along with their interaction. The analysis showed strong evidence for a main effect of sessions ($BF_{10} = 87.061$), indicating better performance in the post-feedback session compared to the pre-feedback session, but inconclusive evidence regarding the feedback type ($BF_{10} = 1.475$). Furthermore, we found evidence supporting a null effect for the interaction between sessions and feedback type ($BF_{10} = 0.336$). In sum, feedback improved performance overall, but its positive effect did not depend on the strategy it was based upon. To explore whether feedback’s effect depended on participants’ strategy, we next investigated the strategies used by participants.

**Table 2:** Summary statistics of behavioral data by feedback and session

Session	Feedback	Resident time	Giving-up time	Number of Patches	Performance	Efficiency
*pre-feedback*						
	FT	12.04(4.58)	3.68(1.11)	16.34(4.69)	46.88(6.19)	.19(0.03)
	GUT	12.37(6.43)	3.83(2.01)	17.10(6.88)	44.39(6.11)	.19(0.02)
*feedback*						
	FT	12.62(2.96)	3.76(0.98)	14.94(2.79)	48.96(4.73)	.20(0.02)
	GUT	13.72(4.30)	4.14(1.34)	14.39(3.88)	47.30(5.10)	.20(0.02)
*post feedback*						
	FT	13.62(3.35)	3.86(1.14)	14.17(3.09)	48.80(5.08)	.20(0.02)
	GUT	13.72(4.34)	4.29(1.23)	14.46(4.16)	47.96(4.29)	.20(0.02)

*Note.* The numbers outside of the brackets are the mean, and the numbers inside the brackets are the standard deviation

#### Strategy analysis

We fitted the computational models of giving-up time (GUT; leaving after consecutive no reward), fixed-time (FT; leaving after staying a predetermined time), and fixed-number (FN; leaving after acquiring a predetermined reward) strategies to participants’ patch-leaving behavior in each session, with the best-fitting model deemed as the strategy used by the participant. These computational models were identical to those used in Lin and von Helversen (2023) and are detailed in Appendix B alongside model and parameter recovery. Table 3 summarizes the number of participants classified as using each strategy. We examined whether strategy use changed from *pre-feedback* to *post-feedback* sessions within each feedback condition using contingency tables and Pearson’s chi-square tests. In the FT feedback condition, we observed a significant change in strategy usage between sessions ($\chi ^2(2) = 7.351, p =.025$.) Conversely, there was no significant difference in strategy usage between sessions when participants received GUT feedback ($\chi ^2(2) = 1.26, p =.532$.) Specifically, while most participants initially relied on GUT strategy in both groups (40 in the FT feedback and 41 in the GUT feedback condition), the number of participants best described by FT strategy increased from 11 to 24 after receiving FT feedback, but not after GUT feedback (from 14 to 12).

**Table 3:** The number of participants using each strategy by session

		Strategy usage			
Feedback	Session	GUT	FT	FN	Total
FT					
	No-feedback	40	11	1	52
	Post-feedback	27	24	1	52
GUT					
	No-feedback	41	14	1	56
	Post-feedback	44	12	0	56

**Table 4:** Participants’ performance and efficiency in the pre- and post-feedback session by strategy and by feedback

	Strategy used in session							
Condition	Pre-feedback	Post-feedback	Performance		Efficiency		N	
FT	FT	FT	44.45 (5.22)	47.00 (2.16)	.19 (.02)	.19 (.01)	11	4
		GUT		47.14 (8.49)		.20 (.03)		7
	GUT	FT	47.7 (6.31)	51.55 (4.62)	.20 (.03)	.21 (.02)	40	20
		GUT		47.25 (3.30)		.20 (.01)		20
GUT	FT	FT	42.79 (6.55)	47.00 (3.71)	.18 (.03)	.20 (.01)	14	7
		GUT		50.14 (4.34)		.21 (.02)		7
	GUT	FT	44.85 (6.00)	46.80 (4.20)	.19 (.02)	.19 (.02)	41	5
		GUT		47.77 (4.43)		.20 (.02)		36

*Note.* The numbers outside of the brackets are the mean, and the numbers inside the brackets are the standard deviation. N refers to the number of participants in the respective cell

To investigate how feedback affected strategy use and performance, we further analyzed participants who used the GUT strategy in the *pre-feedback* session ($N = 88$). As shown in Table 4, among those who received FT feedback ($N = 40$), twenty switched to using the FT strategy. Those who switched showed substantial performance improvement between sessions ($N = 20, BF_{10} = 6.873$), while there was no difference for participants staying with the GUT strategy ($N = 20, BF_{10} = 0.374$). For participants using the GUT strategy in the *pre-feedback* session and receiving GUT feedback (N = 41), most continued to use the GUT strategy in the *post-feedback* session and showed evidence that their performance improved between sessions ($N = 36, BF_{10} = 4.092$). To understand why participants’ performance increased while maintaining the GUT strategy, we analyzed their execution of that strategy. We measured how well participants executed the GUT strategy by the precision of the giving-up-time variable, i.e. the absolute deviance between participants’ giving-up-time and the optimal giving-up-time. We found that precision improved from *pre-feedback* to the *post-feedback* session ($BF_{10} = 2.894$.) A linear mixed model, predicting performance with the precision of giving-up-time and session as fixed effects, outperformed a model without the giving-up-time effect ($BF_{10} = 19.526$).

**Fig. 2:**
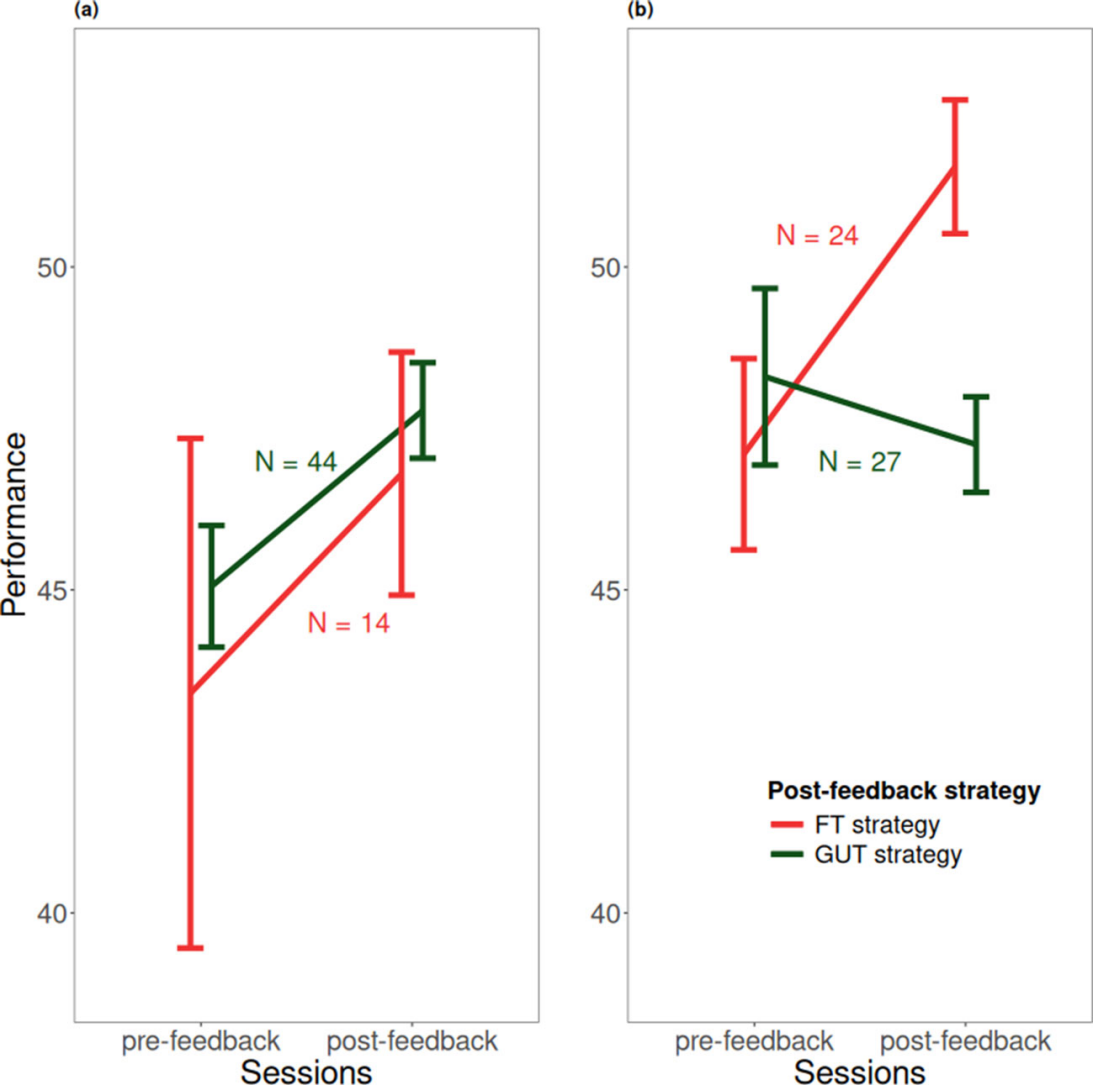
Participants’ performance by session, feedback, and applied strategy. *Note.* The left figure is the performance for the participants who received GUT feedback, and the right figure is the performance for the participants who received FT feedback. The error bar is the standard error

In sum, our results indicate that the feedback provided in the *feedback* session improved participants’ performance in two ways. Feedback from FT led to improvement, encouraging more participants to adopt the FT strategy. Conversely, GUT strategy users benefited from GUT feedback through better execution of the GUT strategy (Fig. 2).

## Discussion

Previous research has indicated that participants tend to prefer the GUT strategy in patch-leaving tasks, even if it results in suboptimal performance. However, even without inducing a strategy switch, an improved execution of the GUT strategy can enhance performance. Corresponding to previous research on the benefit of feedback (Evans & Brown, 2017), we found that providing participants feedback about the appropriateness of their resident time improved performance. Evans and Brown (2017) provided trial-by-trial feedback regarding the correctness and the performance, as well as cognitive feedback after each block. Similarly, we provided trial-by-trial feedback regarding success and cognitive feedback after each patch. Both studies found that participants improved their performance just by trial-and-error feedback. In addition, we identified two ways by which performance could be improved depending on the type of cognitive feedback provided. First, providing participants with feedback based on an optimal application of the GUT strategy led to an improvement in the performance of those using the GUT strategy, which we attribute to better execution of the GUT strategy. Second, feedback based on the (superior) FT strategy prompted a significant number of participants to switch their strategy, leading to enhanced performance during the *post-feedback* phase. In contrast, participants who received FT feedback but kept applying the GUT strategy in the *post-feedback* session ($N=20$) did not improve their performance. This suggests that the performance improvement observed in participants who switched to the FT strategy and those who received GUT feedback cannot be attributed solely to practice effects, as all participants had a similar amount of practice across the three sessions. Only the participants who received feedback that aligned with their strategy used in post-feedback sessions, either because they used the same strategy earlier or switched to a new strategy, improved their performance. Thus, while previous studies showed that most participants used the GUT strategy even in environments where the GUT strategy performs worse than the FT or FN strategies (Hutchinson et al., 2008; Lin & von Helversen, 2023), our results indicate that at least some participants are capable of switching from the GUT strategy to the FT strategy when provided with the appropriate information. These findings suggest that individuals can adopt alternative patch-leaving strategies, such as the FT strategy, and are willing to do so with the appropriate information. They highlight the importance of providing the necessary information to facilitate adaptive strategy switching in the patch-leaving task. Our results, however, also raise the question: what information is required to trigger strategy switching, and is external feedback necessary for it?

Previous research has suggested that without feedback, participants tend to stick with the GUT strategy even if it underperforms compared to simpler strategies (Lin & von Helversen, 2023; Hutchinson et al., 2008). Simulation studies show that the key factor influencing strategy performance is the variance of patch quality (Iwasa, Higashi, & Yamamura, 1981; Hutchinson et al., 2008; Green, 1980), with the FT strategy performing better under low variance while the GUT strategy excels when variance in patch quality is high. Lin and von Helversen (2023) observed that although the GUT strategy may not be optimal in all environments, it performs consistently regardless of the variance of patch quality. This consistent performance makes the GUT strategy a reliable choice under uncertain conditions. In addition, patch quality in a natural environment typically shows an aggregated distribution (Kretzschmar & Adler, 1993), so adapting the GUT strategy as a default strategy would be suitable for ecological reasons. Feedback based on the FT strategy emphasizes that spending equal time at each patch can be beneficial and therefore may help participants recognize when it is appropriate to switch strategies. However, without feedback, participants may struggle to be certain enough about the environment and thus persist with the GUT strategy. In this vein, previous research has not found a switch to the FT strategy even after participants have been exposed to a considerable number of patches (e.g., around 30, Lin & von Helversen, 2023; Hutchinson et al., 2008). Thus, for participants to switch strategies without external feedback that highlights the best strategy, even longer or repeated learning phases may be necessary.

Another possible explanation is that participants did not switch to the FT strategy because they perceived a lack of marginal gain. Adopting a new strategy entails additional cognitive costs (Payne, Bettman, & Johnson, 1988), which may not outweigh the extra effort required. Participants who employed the GUT strategy might have been aware that applying the FT strategy would result in better performance once they began learning about the variance in the environment. However, switching to the FT strategy requires participants to identify the proper resident time to surpass the GUT strategy. This identification process requires additional time, which may not be considered worthwhile compared to the potential performance gain. Consequently, participants opted to stick with the GUT strategy. The FT feedback we provided significantly reduced the time needed to find the proper resident time, making strategy switching more appealing for the participants. It would be interesting to see whether other manipulations that facilitate estimating the optimal threshold for the FT strategy would also foster switching behavior.

Of course, our study also has some limitations that affect the scope of interpretation of our results. For one, it is possible that the participants combined or switched strategies within a session, but we only identified one single strategy per session. We calculated AIC weight to investigate the likelihood of mixing different strategies and found that, especially in the *post-feedback* session, the identified strategy is likely to be the one participants applied; see Appendix B for more detail. Second, we could not test the effect of feedback on the participants who initially used the FT strategy since we only included the participants who used the GUT strategy initially for the important analyses. We planned to exclude those participants before experimenting because prior studies showed that only 20% of the participants used the FT strategy without any intervention. Including those participants would require us to recruit a much larger number of participants. Lastly, while we could compare participants’ performance depending on the strategy feedback, a control group without feedback would allow a more general estimation of the effect of feedback.

While participants who switched from the GUT to the FT strategy in the FT feedback condition showed performance improvement post-switch, we found no overall difference between the FT and GUT feedback conditions. This was likely due to participants in the GUT feedback condition also improving their performance without switching strategies, as the feedback helped them perform the GUT strategy more accurately. Additionally, only 50% of original GUT users switched to the FT strategy in the FT feedback condition; those who did not switch did not benefit from the feedback. Thus, when deciding how to support foraging decisions with feedback, it seems important to consider whether feedback should enable participants to switch to the best strategy or if a better execution of an already known strategy is sufficient.

## Figures and Tables

**Fig. 3:**
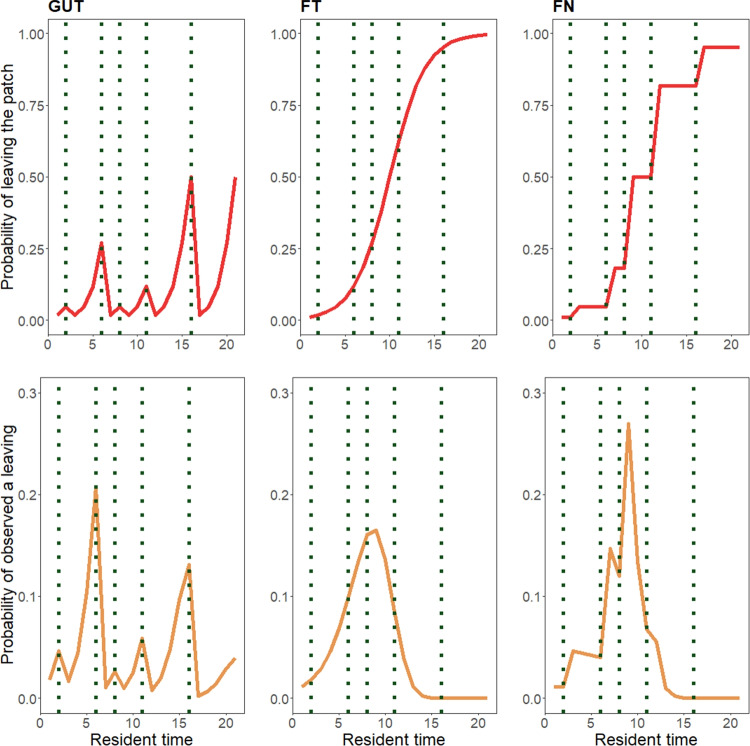
Examples for the behavior predicted by the strategies under an example patch. *Note*. The columns from left to right indicate the behavior predicted by the GUT strategy, the FT strategy, and the FT strategy. The *top row* indicates the probability of leaving the current patch at a certain resident time. The *bottom row* indicates the probability of observing a leaving behavior at a certain resident time. Within each figure, the *x*-axis is the resident time, and the *y*-axis is either the probability of leaving the patch or the probability of observing a leaving behavior. The *green dotted lines* are the reward encounter

**Fig. 4:**
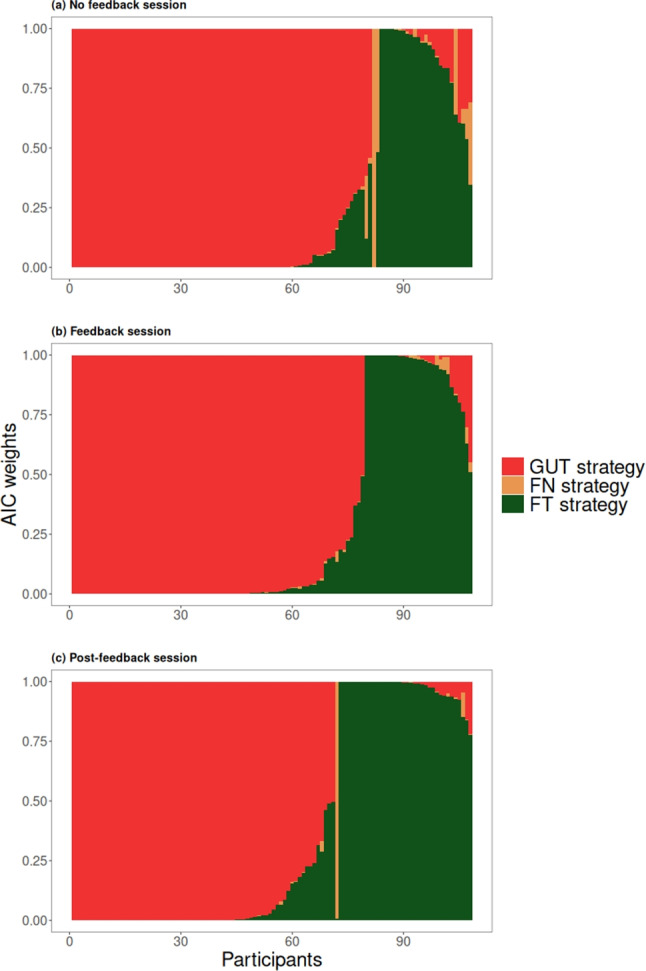
The AIC weights for each participant across sessions. *Note.* The *y*-axis is AIC weights across three strategies. Each *bar* on the *x*-axis represents a participant. The participants are organized by the strategy identified and from the highest AIC weight for the identified strategy to the lowest AIC weight. The *top*, *middle*, and *bottom* figures show the AIC weights for *pre-feedback*, *feedback*, and *post-feedback* sessions, respectively

**Fig. 5:**
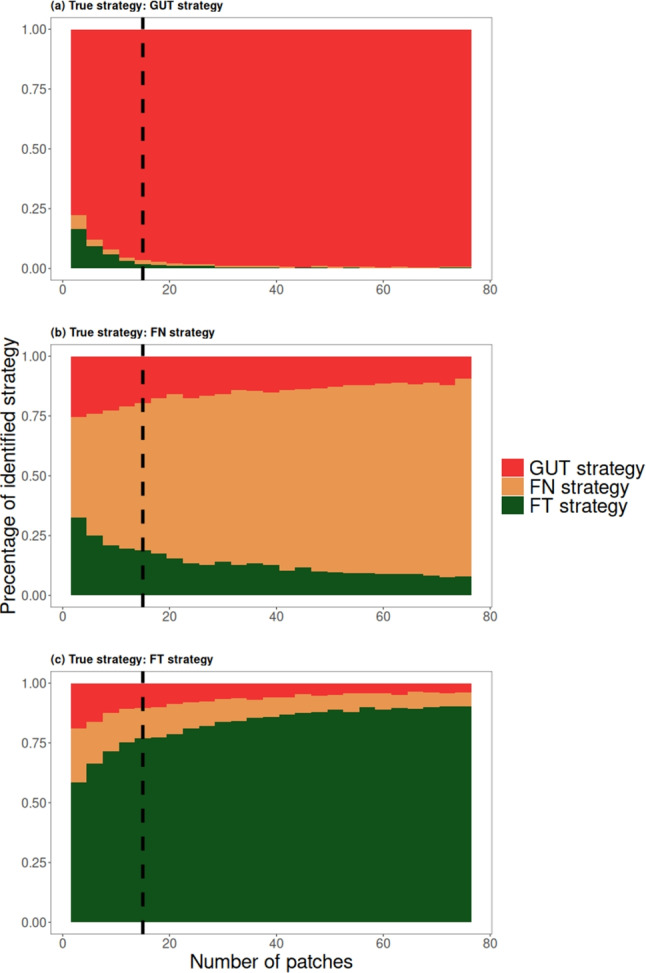
The percentage of identified strategy from different underlying true models across numbers of patches. *Note.* The *x*-axis is the number of patches observed, and the *y*-axis is the percentage of identified strategies. The *dashed line* indicates the average number of patches observed in the experiment. The *top*, *middle*, and *bottom* figures show the identified strategy based on the behavior of the GUT, FN, and FT strategies, respectively

**Fig. 6:**
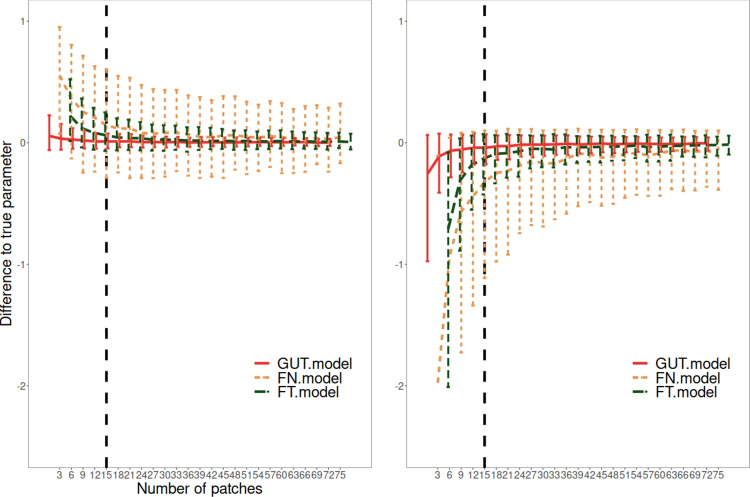
The precision of successfully recovering the underlying parameters. *Note.* The *x*-axis is the number of patches observed, and the *y*-axis is the difference between the estimated parameter and the true parameter. The *left figure* shows the recovery of the decision threshold ($\theta$) for each model, and the *right figure* shows the steepness (*k*) in the logistic function. The *dashed line* indicates the average number of patches observed in the experiment

## Data Availability

The datasets generated during and analyzed during the current study are available in the Open Science Framework repository, https://osf.io/krdq6/?view_only=4fc60ac351cb41a89957cba8c7446510

## Appendix A: Extra analysis

### Utility analysis

We analyzed the reported usefulness of the feedback in a between-subject ANOVA with the strategy used in the *pre-feedback* session and the feedback type as factors. We found evidence for null effects for both main effects and their interaction (strategy used: $BF_{10} = 0.227$; Feedback type: $BF_{10} = 0.252$; Interaction: $BF_{10} = 0.270$). Pair-wise comparison between the participants who changed their strategy from *pre-feedback* to *post-feedback* session and the participants who did not change their strategy revealed indecisive evidence ($BF_{10} = 0.781$). Accordingly, although both FT and GUT feedback improved participants’ performance, participants only reported average levels of usefulness (see Table 5).

**Table 5:** The reported usefulness of feedback based on the type of feedback and the strategy used in the *pre-feedback* session

	Strategy in *no-feedback* session	
Feedback	GUT strategy	FT strategy
FT	2.68(0.97)	2.91(1.22)
GUT	2.83(1.13)	2.92(1.21)

*Note.* The numbers outside of the brackets are the mean, and the numbers inside the brackets are the standard deviation

### Efficiency analysis

We analyzed the efficiency by running a Bayesian ANOVA with the factors sessions (within participants) and feedback (between participants) and their interactions. The analysis showed a strong main effect of sessions ($BF_{10} = 469.657$) but was indecisive in regard to the feedback ($BF_{10} = 1.179$). For the interaction between session and feedback, we found evidence supporting a null effect ($BF_{10} = 0.336$).

For the participants who used the GUT strategy in the *pre-feedback* session and received FT feedback, the participants who switched to the FT strategy ($N = 20$) showed substantial improvement in efficiency ($BF_{10} = 15.406$), but we found no difference for the participants who stayed with the GUT strategy ($N = 20$, $BF_{10} = 0.303$). For the participants who used the GUT strategy in both *pre-feedback* and *post-feedback* sessions and received GUT feedback ($N = 41$), we found a marginal improvement in the participants’ efficiency between sessions ($BF_{10} = 2.842$).

### Exploratory analysis

We analyzed whether the participants’ performance in the *no-feedback* or the *feedback* session affects whether the participants switch their strategy between the *no-feedback* and the *post-feedback session*, with the hypothesis that the participants who performed poorly could be more incentivized to switch their strategy. We first only analyzed the participants who received the FT feedback and used the GUT strategy in the *no-feedback* session, as those participants were the ones who showed most of the switches, with Bayesian logistic regression through brm function in arms package (Bürkner, 2017). We found evidence against a correlation between the performance in the *no-feedback* and the *feedback* condition with strategy change ($BF_{10}=0.161$ for *no-feedback session* and $BF_{10}=0.170$ for *feedback session*). Including the participants that received GUT feedback showed similar findings; we found evidence against the correlation between performance in both the *no-feedback* and the *feedback* sessions and strategy change ($BF_{10}=0.098$ for *no-feedback* and $BF_{10}=0.120$).

## Appendix B: Strategy analysis

To find out the strategy used by the participants for their patch-leaving decision, we implemented the same computational model as in Lin and von Helversen (2023). We present the details of the modeling process in this session. The models for GUT, FT, and FN strategies shared the same basic structure as

1 $$\begin{aligned} P_{leave}(t|\theta ) = \frac{1}{1 + \exp \{ -k\cdot [factor(t)-\theta ] \}}. \end{aligned}$$

The $P_{leave}(t|\theta )$ is the probability of leaving at time step $t$ with the parameter $\theta$ and was determined though a logistic function with the parameter $\theta$ as the sigmoid midpoint and the $k$ as the steepness of the curve. The $factor(t)$ is the decision factor used by the decision strategy at time step $t$, and different models had different decision factors. The decision factor for the GUT strategy is the accumulated time steps of getting no reward. For the FT and the FN strategies, the decision factor is the resident time units and the number of rewards acquired, respectively. The top row of Fig. 3 is an example of $P_{leave}(t|\theta )$ within a patch under different decision strategies.

The $P_{leave}(t|\theta )$ is the probability of leaving the current patch at certain time $t$ regardless of previous status. However, to actually observe a leaving decision at time $t$, the participants had to stay within the patch until time $t-1$, and the probability of staying time $t-1$ is the conjunction probability of not leaving at previous time steps, $\prod _{i=1}^{t-1}[1-P_{leave}(i|\theta )]$. The likelihood of observing a leaving decision at time $t$ is the conjunction probability of leaving at time $t$, $P_{leave}(t|\theta )$, and the probability of staying until time $t-1$, as

2 $$\begin{aligned} likelihood(t|\theta ) = \prod _{i=1}^{t-1} [1-P_{leave}(i|\theta )] \cdot P_{leave}(t|\theta ). \end{aligned}$$

The likelihood was demonstrated in the bottom row of Fig. 3. Once we calculated the likelihood of observing a leaving decision at a certain time step, we maximized the sum of the log-likelihood across all the data points within a session per participant. The maximization was done through the Differential Evolution algorithm in DEoptim library (Ardia, Boudt, Carl, Mullen, & Peterson, 2011) in R. We then calculated the Akaike information criterion, AIC, as

3 $$\begin{aligned} AIC = -2 \times \sum _{l=1}^{n} log[likelihood_l(\theta )] + 2 \cdot N, \end{aligned}$$

where the $N$ is the number of parameters in the model, and the $n$ is the number of leaving decision observed from a participant within a session. The strategy used by the participants was assumed to be the model with the lowest AIC. To examine the likelihood of successfully identifying the strategy used by the participants, we calculated the AIC weights (Wagenmakers & Farrell, 2004) for each participant at each session, as shown in Fig. 4. Overall, the strategy identification in *pre-feedback* session showed some ambiguity. However, the FT strategy identified in *post-feedback* session was pretty successful, while some GUT strategies identified were ambiguous.

In addition, we ran a model recovery test to ensure that we were successful in identifying the strategy used by the participants. We first generated the environment according to the experiment design, with each river quality sampled equally. The number of rivers ranged from 3 to 75 with step of 3 to ensure all the river qualities had even distribution. We then simulated leaving behaviors from each model with the model parameters randomly selected from the parameters estimated from the participants’ data. The simulated leaving behaviors were then fitted with the same process as the strategy analysis. The rate of successfully recovering the underlying parameters was shown in Fig. 5. On average, the number of rivers the participants experienced was 15.4 rivers per session. Hence, the successful recovery rate was 96.50%, 77.10%, and 61.56% on average. We also plot the difference between the underlying and estimated parameters in Fig. 6.

## Footnotes

1 How the resource depletes also affects the performance of FN and FT. If the resource depletes based on the time spent in the patch, the FT strategy tends to outperform FN. If the resource depletes based on the number of rewards acquired, the FN strategy outperforms FT. The depletion factor, however, has less impact than the patch quality variance. See Table 7 of Lin and von Helversen (2023) for more detail.

2 The data and materials are available at Open Science Framework https://osf.io/krdq6/?view_only=4fc60ac351cb41a89957cba8c7446510, and Experiment was preregistered on AsPredicted https://aspredicted.org/5PJ_36R.

3 The participants were asked to rate the utility of the feedback in a five-point Likert scale between 1 (most useless) to 5 (most useful). The results, however, suggest no differences between the conditions. We report the analysis in Appendix A.

4 Since excluding the last river affects the time horizon available for each participant, we also tested another variable, efficiency, to measure performance. Efficiency was defined as the ratio between the amount of gold acquired (excluding the last river) and the total amount of time available (excluding the last river). We found a similar pattern of results for efficiency as for the total amount of gold. The results for efficiency are reported in Appendix A.

## Code Availability

The experiment and the analysis script for the current study are available in the Open Science Framework repository, https://osf.io/krdq6/?view_only=4fc60ac351cb41a89957cba8c7446510

## References

[CR1] Ardia, D., Boudt, K., Carl, P., Mullen, K., & Peterson, B. G. (2011). Differential evolution with DEoptim: An application to non-convex portfolio optimization. *the R Journal,**3*(1), 27–34.

[CR2] Bettinger, R. L., & Grote, M. N. (2016). Marginal value theorem, patch choice, and human foraging response in varying environments. *Journal of Anthropological Archaeology,**42*, 79–87. 10.1016/j.jaa.2016.03.002

[CR3] Bürkner, P.-C. (2017). Brms: An r package for bayesian multilevel models using stan. *Journal of Statistical Software,**80*, 1–28. Retrieved April 1, 2025, from https://www.jstatsoft.org/article/view/v080i01/0

[CR4] Charnov, E. L. (1976). Optimal foraging, the marginal value theorem. *Theoretical Population Biology,**9*(2), 129–136. Publisher: Elsevier. 10.1016/0040-5809(76)90040-x 1273796

[CR5] Constantino, S. M., & Daw, N. D. (2015). Learning the opportunity cost of time in a patch-foraging task. *Cognitive, Affective, & Behavioral Neuroscience,**15*(4), 837–853. Publisher: Springer. 10.3758/s13415-015-0350-y PMC4624618 25917000

[CR6] De Leeuw, J. R. (2015). jsPsych: A JavaScript library for creating behavioral experiments in a web browser. *Behavior Research Methods,**47*(1), 1–12. 24683129 10.3758/s13428-014-0458-y

[CR7] Evans, N. J., & Brown, S. D. (2017). People adopt optimal policies in simple decision-making, after practice and guidance. *Psychonomic Bulletin & Review,**24*(2), 597–606. 10.3758/s13423-016-1135-1 27562760 10.3758/s13423-016-1135-1

[CR8] Green, R. F. (1980). Bayesian birds: A simple example of oaten’s stochastic model of optimal foraging. *Theoretical Population Biology,**18*(2), 244–256. Publisher: Elsevier.

[CR9] Green, R. F. (1984). Stopping rules for optimal foragers. *The American Naturalist,**123*(1), 30–43. Publisher: University of Chicago Press.

[CR10] Hassall, C. D., & Krigolson, O. E. (2020). Feedback processing is enhanced following exploration in continuous environments. *Neuropsychologia,**146*, 107538. 10.1016/j.neuropsychologia.2020.107538 32574615 10.1016/j.neuropsychologia.2020.107538

[CR11] Hutchinson, J. M., Wilke, A., & Todd, P. M. (2008). Patch leaving in humans: Can a generalist adapt its rules to dispersal of items across patches? *Animal Behaviour,**75*(4), 1331–1349. Publisher: Elsevier.

[CR12] Iwasa, Y., Higashi, M., & Yamamura, N. (1981). Prey distribution as a factor determining the choice of optimal foraging strategy. *The American Naturalist,**117*(5), 710–723. Publisher: University of Chicago Press.

[CR13] Krebs, J. R. (1973). Behavioral aspects of predation. In P. P. G. Bateson & P. H. Klopfer (Eds.), *Perspectives in ethology* (pp. 73–111). 10.1007/978-1-4615-7569-6_3

[CR14] Kretzschmar, M., & Adler, F. R. (1993). Aggregated distributions in models for patchy populations. *Theoretical Population Biology,**43*(1), 1–30. 10.1006/tpbi.1993.1001 8451752 10.1006/tpbi.1993.1001

[CR15] Lin, H.-Y., & von Helversen, B. (2023). Never gonna give you up even when it is suboptimal. *Cognitive Science,**47*(7), e13323. _eprint: https://onlinelibrary.wiley.com/doi/pdf/10.1111/cogs.13323. 10.1111/cogs.13323 10.1111/cogs.13323 37486808

[CR16] McNair, J. N. (1982). Optimal giving-up times and the marginal value theorem. *The American Naturalist,**119*(4), 511–529. Publisher: University of Chicago Press.

[CR17] Morey, R. D., & Rouder, J. N. (2015). BayesFactor: Omputation of bayes factors for common designs (Version R package version 0.9.12-2). Retrieved from https://CRAN.R-project.org/package=BayesFactor

[CR18] Newell, B. R., & Rakow, T. (2007). The role of experience in decisions from description. *Psychonomic Bulletin & Review,**14*(6), 1133–1139. 10.3758/BF03193102 18229486 10.3758/bf03193102

[CR19] Pacheco-Cobos, L., Winterhalder, B., Cuatianquiz-Lima, C., Rosetti, M. F., Hudson, R., & Ross, C. T. (2019). Nahua mushroom gatherers use area-restricted search strategies that conform to marginal value theorem predictions. *Proceedings of the National Academy of Sciences,**116*(21), 10339–10347. Publisher: National Acad Sciences. 10.1073/pnas.1814476116 PMC6535025 31061117

[CR20] Payne, J. W., Bettman, J. R., & Johnson, E. J. (1988). Adaptive strategy selection in decision making. *Journal of Experimental Psychology: Learning, Memory, and Cognition,**14*(3), 534. Publisher: American Psychological Association.

[CR21] Pyke, G. H. (1978). Optimal foraging in hummingbirds: Testing the marginal value theorem. *American Zoologist,**18*(4), 739–752. Publisher: Oxford University Press UK.

[CR22] R Core Team. (2024). *R: A language and environment for statistical computing*. Vienna, Austria: R Foundation for Statistical Computing. Retrieved from https://www.R-project.org/

[CR23] Starns, J. J., & Ratcliff, R. (2010). The effects of aging on the speed-accuracy compromise: Boundary optimality in the diffusion model. *Psychology and Aging,**25*(2), 377–390. 10.1037/a0018022 20545422 10.1037/a0018022 PMC2896207

[CR24] van Alphen, J. J. M., Bernstein, C., & Driessen, G. (2003). Information acquisition and time allocation in insect parasitoids. *Trends in Ecology & Evolution,**18*(2), 81–87. 10.1016/S0169-5347(02)00035-6

[CR25] Wagenmakers, E.-J., & Farrell, S. (2004). AIC model selection using akaike weights. *Psychonomic Bulletin & Review,**11*(1), 192–196. 10.3758/BF03206482 15117008 10.3758/bf03206482

[CR26] Wilke, A., Hutchinson, J. M. C., Todd, P. M., & Czienskowski, U. (2009). Fishing for the right words: Decision rules for human foraging behavior in internal search tasks. *Cognitive Science,**33*(3), 497–529. _eprint: https://onlinelibrary.wiley.com/doi/pdf/10.1111/j.1551-6709.2009.01020.x. 10.1111/j.1551-6709.2009.01020.x 10.1111/j.1551-6709.2009.01020.x 21585478

[CR27] Wolfe, J. M. (2013). When is it time to move to the next raspberry bush? foraging rules in human visual search. *Journal of Vision,**13*(3), 10–10. Publisher: The Association for Research in Vision and Ophthalmology. 10.1167/13.3.10 10.1167/13.3.10 PMC4521330 23641077

[CR28] Zhang, J., Gong, X., Fougnie, D., & Wolfe, J. M. (2015). Using the past to anticipate the future in human foraging behavior. *Vision Research,**111*, 66–74. Publisher: Elsevier. 10.1016/j.visres.2015.04.003 PMC4442738 25872176

[CR29] Zhang, J., Gong, X., Fougnie, D., & Wolfe, J. M. (2017). How humans react to changing rewards during visual foraging. *Attention, Perception, & Psychophysics,**79*(8), 2299–2309. 10.3758/s13414-017-1411-9 10.3758/s13414-017-1411-9 28856626

